# Fellow-Workers and the Roll of Honour

**Published:** 1915-04-10

**Authors:** 


### ROUND THE WORLD.

[The Editor invites Early Information and Contributions Marked "Fellow-Workers."]

### Fellow-Workers and the Roll of Honour.

The dental department at the King George Hospital has been placed in charge of the following well-known experts, all of whom hold a surgical diploma in addition to their dental qualification: Mr. Frederick Walter Barrett; Mr. William A. Maggs, consulting surgeon to the dental department at Guy's Hospital; Mr. Morton Smale, consulting surgeon to St. Mary's Hospital, and a prominent consultant of the Royal Dental Hospital, where he was formerly dean; Mr. Sidney Spokes, consulting surgeon to the National Dental Hospital; Mr. Arthur Underwood, formerly professor of dental surgery at King's College; and Mr. Charles Edward Wallis, dental surgeon to King's College Hospital. The British Dental Association is making a donation to the expenses of this important department.

The anæsthetic staff appointed to the same institution includes Dr. G. A. H. Barton, of the Golden Square Throat Hospital, the Royal National Orthopædic Hospital, and the Hampstead General Hospital; Dr. N. Whitelaw Bourns, of Westminster Hospital; Mr. C. Carter Braine, of Charing Cross and St. Peter's Hospitals; Mr. J. H. Chaldecott, of St. Mary's; Mr. Herbert Charles, of Middlesex Hospital; Dr. C. J. Loosely, of the Great Ormond Street, Metropolitan Ear, Nose and Throat, and Royal Dental Hospitals; Sir Frederick Hewitt, M.V.O.; Dr. Richard Gill, chief chloroformist to St. Bartholomew's; Mr. Harvey Hilliard, of the French and Royal Dental Hospitals; Dr. Harold Low, of St. Thomas's; and Sir John McCall. Also Dr. Vivian B. Orr, of the Westminster and Royal Free Hospitals; Mr. H. Marmaduke Page, of Guy's and the West London Hospitals; Dr. H. R. Phillips, of the Italian Hospital and the West End Hospital for Nervous Diseases; Dr. Llewelyn Powell, of St. George's Hospital, and the National Hospital for Paralysis and Epilepsy; Dr. W. E. Robinson, of the West London and Royal Waterloo Hospitals; Mr. George Rowell, senior anæsthetist to Guy's; Dr. Francis E. Shipway, also of Guy's; and Mr. C. H. C. Visick, of the Great Northern Central and Hampstead General Hospitals.

Sir Frederick Hewitt, M.V.O., occupies a unique position, and has received honours that are rarely won by medical men taking up anæsthetics as a specialty. Physician anæsthetist to St. George's Hospital, he is also consulting anæsthetist and emeritus lecturer on anæsthetics to the London Hospital, which institution he served as a member of the active staff for many years. It fell to Sir Frederick to give the anæsthetic when the late King was stricken down with acute appendicitis, and he occupies the post of anæsthetist on the establishment of his Majesty King George V. Qualifying M.R.C.S., L.S.A., in 1882, he subsequently became an M.D. Cantab., and in the course of a busy life has held important appointments at Charing Cross, the Royal Dental, and the Royal Waterloo Hospitals, in addition to those already mentioned.

Lieutenant-Colonel Charles Boyce, who is in charge of the Hôpital Militaire Anglais at Nevers, served in the Territorial department of the R.A.M.C., in which he reached his present rank, for many years. An M.D. Edin. who qualified in 1876, he was at one time honorary physician to the West Kent Hospital at Maidstone, for which borough he is a Justice of the Peace.

Sir Lambert Hepenstal Ormsby is to be congratulated on his appointment as consulting surgeon to the New Zealand Expeditionary Force, in which capacity he has been granted the rank of lieutenant-colonel. One of the most distinguished Irish surgeons of the day, holding the post of senior surgeon to the Meath Hospital in Dublin, Sir Lambert, who was, indeed, born in New Zealand, was formerly President of the Royal College of Surgeons of Ireland, as well as examiner in surgery for the War Office. He qualified at Dublin in 1869, subsequently taking the M.D. degree and becoming an F.R.C.S.I.

The assistant medical officer of health of Liverpool, Dr. Arthur Augustus Mussen, is joining the staff of the Merchants' Hospital. A graduate of Dublin, where he took his M.D. in 1895, Dr. Mussen studied also at University College, London, and took the D.P.H. of Cambridge in 1899. After qualifying he was for a time house physician to the Hospital for Women in Soho Square, and went to Liverpool as a resident at the Northern Hospital. Then, taking up public health work, he held the position of assistant medical officer of health at Huddersfield before taking up his present appointment at Liverpool, where he is also lecturer and examiner on his special subject at the University.

Dr. Harold Andrew Kidd, who will be given military rank as administrator of the Graylingwell Mental Hospital at Chichester when the War Office takes it over for the wounded, is the medical superintendent of that institution. A St. Mary's man, who qualified L.R.C.P., M.R.C.S. in 1889, Dr. Kidd was at one time senior assistant medical officer at Claybury.

At Graylingwell Mental Hospital, which is the West Sussex County Council's Asylum, it is anticipated that 1,000 soldiers will be accommodated, its present staff being retained. The late Mr. F. A. K. Stuart, who lost his life recently through a motor accident, was one of the two assistant medical officers at Chichester, the other being Dr. G. E. Peachell, a St. Mary's man, who qualified M.B., B.S. Lond. with honours in 1904, and took the M.D. Lond. in psychiatry in 1912.

Another medical officer of health who is now serving with the Army is Dr. Arthur Edward Jerman, who has been looking after the welfare of Erith. Dr. Jerman, who has recently been promoted to the rank of lieutenant-colonel in the R.A.M.C. (T.), entered Westminster Hospital Medical School with a scholarship, and qualifying L.R.C.P., M.R.C.S. in 1896, subsequently became an M.B. Lond., D.P.H. Oxon. Later he was for a time house surgeon at Westminster, and worked in the Colonies as medical officer in Uganda. On the outbreak of war he mobilised as major in the R.A.M.C. (T.) attached to the 4th Field Ambulance of the 2nd London Division.

